# An integrated genetic algorithm-machine learning approach for morphological optimization of high-rise residential districts in Yulin

**DOI:** 10.1371/journal.pone.0330913

**Published:** 2025-09-02

**Authors:** Juan Ren, Yuan Meng, Yu Liu

**Affiliations:** 1 School of Architecture, Xi’an University of Architecture and Technology, Xi’an, Shaanxi, China; 2 School of Architecture, Chang’an University, Xi’an, Shaanxi, China; 3 School of Architecture, Southeast University, Nanjing, Jiangsu, China; 4 School of Mechanics and Tramsportation Civil Engineering and Architecture, Northwestern Polytechnical University, Xi’an, Shaanxi, China; The Chinese University of Hong Kong, HONG KONG

## Abstract

The pursuit of global carbon neutrality necessitates addressing the dual challenge of enhancing solar energy utilization while improving thermal comfort in high-rise residential areas, particularly in Yulin, northern Shaanxi, China, where abundant solar resources exist but maximizing solar acquisition often compromises summer thermal environment quality. This resource-comfort contradiction highlights the need for balanced architectural strategies in regions with pronounced seasonal variations. Building morphological parameter optimization is crucial for balancing annual solar energy capture against summer overheating risks, yet research remains insufficient. This study developed parametric layout models using Rhino-Grasshopper, considering key parameters including building length, width, height, density, floor area ratio, and south-facing angle deviation. Multi-objective optimization was conducted using NSGA-II genetic algorithm under regulatory constraints, while combining traditional regression analysis with convolutional neural networks (CNN) to investigate the influence mechanisms of these morphological parameters. Results indicate that the optimized building morphology can increase annual solar radiation acquisition (SRA) by 2.57% while maintaining comfortable summer Universal Thermal Climate Index (UTCI) values, effectively balancing solar energy capture and outdoor thermal comfort. Regression analysis revealed a positive correlation between building length and summer UTCI (r = 0.73), whereas CNN identified a negative correlation (−0.45). Both methods identified similar parameter combinations affecting SRA, with CNN demonstrating superior capability in capturing complex non-linear relationships. These findings provide evidence-based design guidelines specific to Yulin while offering implications for sustainable residential development in similar climates, advancing the integration of climate-adaptive design strategies.

## 1 Introduction

### 1.1 Research background

As global climate change becomes increasingly severe, countries worldwide have established carbon neutrality targets to achieve sustainable development and ecological balance. The Chinese government has explicitly set the “dual carbon” goals of reaching peak carbon emissions by 2030 and achieving carbon neutrality by 2060 [1,2]. Against this backdrop, effectively utilizing renewable energy, particularly solar energy, has become a crucial challenge in architectural design. As a clean and renewable energy source, solar energy possesses enormous development and utilization potential. Specifically, in architectural design, maximizing solar radiation acquisition (SRA) potential through rational morphological layout and optimized design can not only significantly reduce carbon emissions during building operation but also help alleviate energy constraints to some extent [3].

Simultaneously, with accelerating urbanization and improving residential living standards, outdoor thermal comfort has become a key consideration in current residential district design. Particularly in high-density urban environments, improper building layouts may exacerbate the heat island effect, affecting residents’ health and quality of life. Outdoor thermal comfort is not only closely related to climatic conditions but is also influenced by multiple factors such as building geometric morphology, arrangement patterns, and orientation within residential areas. Therefore, enhancing outdoor thermal comfort through scientifically sound building morphology and layout design has become one of the important research directions.

The Yulin region of Northern Shaanxi, located in China’s inland north western plateau within the temperate monsoon climate zone, is one of China’s high-value solar radiation areas. Its total solar radiation resource reserve reaches 6.13 × 1013 kWh, with utilizable solar radiation resources of 1226 kWh, indicating significant potential value for solar energy utilization [4]. This high solar radiation characteristic provides abundant resources for solar energy utilization while potentially exacerbating outdoor thermal discomfort during summer, presenting a challenge for building morphology optimization design to balance energy acquisition and thermal comfort.

Since building morphology and layout play a decisive role in the acquisition and utilization of solar energy resources, optimizing building geometry, arrangement patterns, and orientation based on local climatic characteristics can not only maximize solar energy capture and utilization but also effectively improve the outdoor thermal environment of residential areas. Unlike previous single-objective optimization or simple linear analysis approaches, this study innovatively uses Yulin region’s high solar radiation characteristics and outdoor thermal comfort requirements as research entry points, adopting multi-objective optimization methods for comprehensive consideration. This research focuses on the morphological layout of high-rise residential areas in northern Shaanxi’s Yulin, aiming to explore the influence mechanisms of building morphological parameters through genetic algorithms, innovatively combining regression analysis and convolutional neural network (CNN) models from machine learning. By integrating Rhino-Grasshopper thermal comfort simulation software and Wallacei multi-objective optimization tools, this study investigates how to achieve dual optimization goals of increasing the total annual solar radiation acquisition on building surfaces while reducing summer outdoor Universal Thermal Climate Index (UTCI) by controlling key parameters such as building length, width, height, southern deviation angle, density, and floor area ratio, while meeting fire safety and sunlight exposure regulatory requirements. Research results demonstrate that the optimized building layout solution maintains favorable summer UTCI levels while increasing annual solar radiation acquisition, achieving building layout optimization under the dual context of carbon neutrality goals and improving residents’ quality of life.

In cold climate regions such as Yulin, while maximizing solar radiation acquisition provides substantial winter heating benefits, it may simultaneously exacerbate summer thermal stress, thus requiring sophisticated multi-objective optimization strategies.

### 1.2 Literature review

Multi-objective optimization of high-rise residential district morphology and layout has been attracting increasing attention in architectural research. Delgarm et al. [5] used a multi-objective artificial bee colony algorithm to optimize office room parameters like orientation, windows, and materials, balancing energy consumption and thermal discomfort. Yi et al. [6] optimized sunlight rights issues in high-rise apartments through the Rhino-Grasshopper platform using genetic algorithms (GA), improving sunlight efficiency by 28.3%. Yang et al. [7] employed Non-Dominated Sorting Genetic Algorithm (NSGA) to optimize building envelope materials, achieving multi-objective trade-offs between energy conservation and environmental quality. Chen et al. [8] combined EnergyPlus with genetic algorithms to optimize building cluster morphology, increasing solar energy potential by 15.7%. Caruso et al. [9] developed a building morphology optimization framework considering multiple constraints, improving natural lighting by 19% while reducing energy consumption. These studies confirm the significant effectiveness of genetic algorithm-based multi-objective optimization in enhancing building performance.

Regarding outdoor thermal comfort research has primarily focused on assessment methods and optimization strategies. Allegrini et al. [10] analyzed the impact of high-rise building cluster morphology on microclimate through CFD simulation, finding a significant negative correlation between building height and UTCI (R² = 0.75). Ventilation strategies have also been shown to significantly affect indoor environmental comfort, a concept that can be extended to building morphology for optimizing outdoor thermal comfort [11]. Zhang et al. [12] established an urban thermal environment assessment model based on measured data, quantifying the impact of building density on UTCI (coefficient 0.68). Yang et al. [13] optimized building layout using parametric methods, achieving an average summer UTCI reduction of 2.4°C. Vallati et al. [14] studied the relationship between building morphology and street canyon thermal environment, providing design strategies for improving thermal comfort. These studies provide scientific basis for improving residential district thermal environments.

In SRA optimization, Compagnon [15] analyzed the impact of building morphology on solar energy potential, establishing a prediction model with 87% accuracy. Khalil et al. [16] optimized open office building form, reducing thermal energy use intensity by 22.76%. Martins et al. [17] proposed solar energy optimization strategies for high-rise buildings, increasing annual power generation efficiency by 18.5%. Hachem et al. [18] systematically studied the relationship between building morphology and SRA, developing solar facade design methods. These studies laid the foundation for improving solar energy utilization efficiency.

Regression analysis in machine learning has been widely applied as an important tool for exploring relationships between building morphology and environmental performance. Ratti et al. [19] revealed correlations between building morphology and energy consumption (R² = 0.83) through machine learning’s multiple regression algorithms. Wong et al. [20] quantified the impact of morphological parameters on natural ventilation using hierarchical regression models in machine learning. Sarralde et al. [21] established prediction models for urban morphology and solar energy potential using supervised learning methods, achieving 85% accuracy. Lin et al. [22] explored the relationship between morphological spatial patterns of green space and urban heat island intensity using machine learning methods, providing insights for morphological optimization in urban building clusters. These studies validate the reliability of machine learning regression models in identifying key influencing factors.

CNN in deep learning has demonstrated unique advantages in building environment optimization. Minassian [23] et al. developed CNN, LSTM, and hybrid CNN-LSTM models for indoor temperature prediction in smart buildings, with multivariate inputs enhancing prediction accuracy. Lei [24] applied a deep meta-learning model based on CNN-LSTM to predict outdoor thermal comfort, improving prediction accuracy and thermal environment performance in urban districts. Yang et al. [25] conducted a comprehensive review of occupancy sensing systems and modeling methodologies, highlighting machine learning approaches that achieved substantial accuracy improvements for building performance assessment and energy management. Natanian et al. [26] combined deep learning CNN with parametric design to optimize building cluster morphology and energy performance. These studies confirm the superiority of machine learning methods in complex building environment analysis.

**Fig 1:**
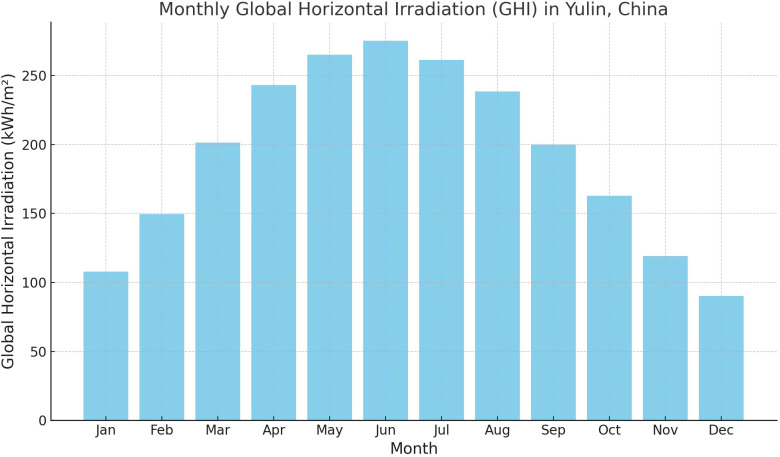
Monthly Distribution of Global Horizontal Irradiance (GHI) in Yulin Region (Data Source: https://energyplus.net/weather-search/yulin).

**Fig 2:**
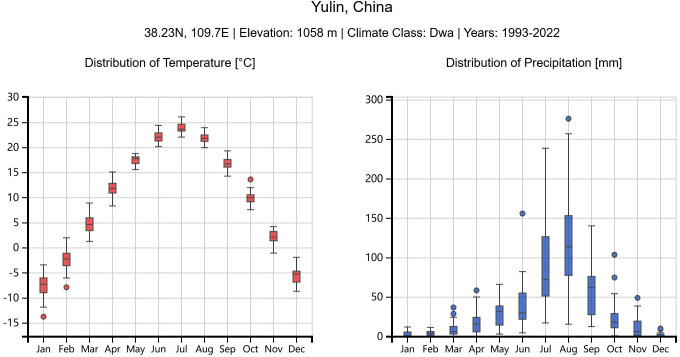
Monthly Statistical Distribution of Temperature (Left) and Precipitation (Right) in Yulin Region [28].

In optimizing high-rise residential district morphology and layout in Yulin, accurately understanding its climatic characteristics is crucial for SRA and summer thermal environment improvement. Located in Northern Shaanxi, China, Yulin has a temperate continental monsoon climate with abundant solar energy resources but significant summer thermal environment challenges. According to meteorological data [27], Fig 1 shows the monthly Global Horizontal Irradiation (GHI) distribution in Yulin. The GHI peaks between May and August, particularly in June and July, reaching approximately 275.1 kWh/m² and 261.4 kWh/m² respectively. From Fig 2, it can be observed that the Yulin region maintains moderate overall temperatures during summer, with generally good thermal comfort conditions and relatively favorable thermal comfort levels [28]. However, a singular pursuit of maximizing solar radiation acquisition could potentially disrupt the current summer thermal comfort status. The coexistence of high solar radiation and thermal comfort demands makes Yulin a typical case study for researching the impact of building morphology. Although Yulin’s summer thermal environment is relatively manageable compared to other extreme climate regions, in-depth research on the influence of building morphology on thermal comfort holds triple value in the context of intensifying global climate change: first, it can establish optimal reference standards for building morphology design in this region; second, the developed multi-objective optimization methods can provide methodological support for regions with similar climatic characteristics; finally, morphological optimization that enhances summer ventilation performance can simultaneously improve winter microclimate conditions, achieving comprehensive benefits of year-round climate adaptability.

For high-rise residential districts, summer diurnal temperature variation (averaging 12–14°C) and prevailing wind direction (northwest wind) significantly influence district layout optimization [29]. Building cluster morphology layout in high-rise residential districts must meet environmental requirements for different seasons: in summer, reasonable layout is needed to create shading effects and guide airflow organization to improve outdoor thermal environment; year-round optimization of building orientation and spacing is required to ensure adequate solar radiation for the district. This seasonal variation in requirements presents challenges in trade-offs and balance for high-rise residential district morphology and layout optimization.

In summary, previous research has made significant progress in multi-objective optimization of high-rise residential district morphology and layout, UTCI, SRA, and machine learning applications. However, there is limited research focusing on climate regions like Yulin, which is characterized by abundant solar energy resources, arid and large temperature variations. Furthermore, existing studies predominantly focus on single optimization techniques, with limited exploration of the synergistic application of genetic algorithms, regression analysis, and CNN methods. Particularly at the morphological layout design level of high-rise residential districts in Yulin, the question of how to maximize solar energy acquisition while simultaneously improving the summer outdoor thermal environment has not been thoroughly investigated or definitively answered.

### 1.3 Research objectives

This study employs an integrated approach combining Rhino-Grasshopper thermal comfort simulation software and the Walacei multi-objective optimization tool to conduct a comprehensive analysis and optimization of high-rise residential district morphology in Yulin. The primary objective is to achieve an optimal balance between enhancing outdoor thermal comfort in summer and maximizing annual solar radiation on building surfaces, while adhering to fire safety and solar access regulations. This is accomplished through the optimization of key morphological parameters including building geometry (length, width, height), southern orientation angle, building density, and floor area ratio. Furthermore, both regression analysis and CNN are employed to analyze the data generated during the optimization process, investigating the underlying mechanisms by which morphological parameters influence the two objective values. By revealing the intrinsic relationships between local high-rise residential district morphology, summer outdoor thermal comfort, and annual solar radiation, this study aims to provide designers with more scientific and efficient design strategies while contributing theoretical support and practical guidance to related fields.

## 2 Research methods

### 2.1 Parametric and performance model construction

#### 2.1.1 Building parameters.

Parametric modeling and performance simulation are crucial steps in achieving multi-objective optimization. Rhino and Grasshopper are employed as design and simulation software in this study, integrated with the Wallacei plugin, to construct detailed building models [30,31].

**Fig 3:**
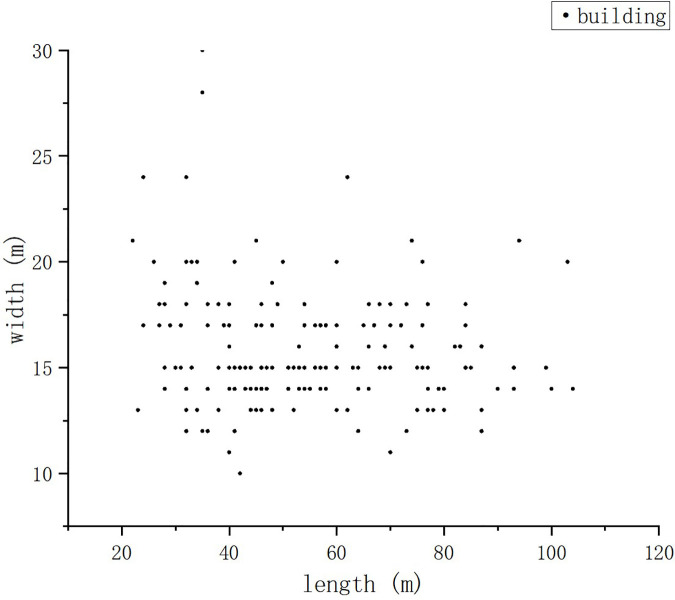
The length and width of main residential buildings in Yulin.

Based on comprehensive field surveys of 115 typical high-rise residential districts in Yulin [32], statistical analysis reveals that 76.5% adopt parallel layouts and 18.3% use staggered arrangements, both conforming to rectangular grid characteristics and representing 94.8% of local developments. The parallel and staggered layouts are identified as prevalent configurations in this region (Fig 3).

**Fig 4:**
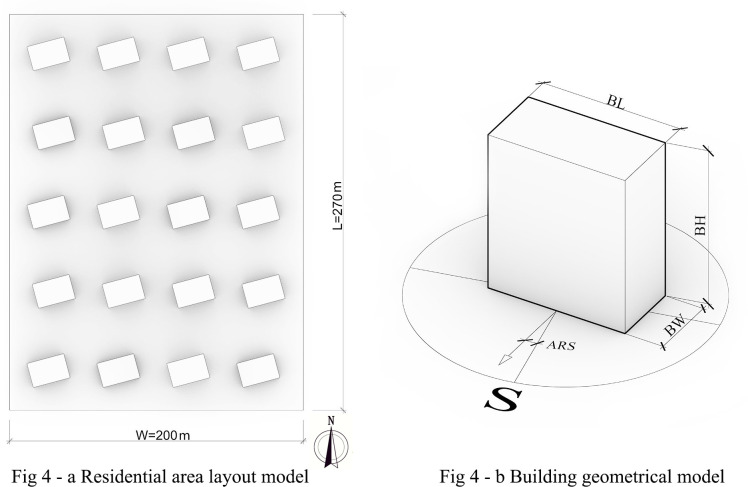
Schematic of basic architectural layout morphological parameter modeling.

This study adopts a validated baseline parametric layout model: the site dimensions are set at 270 m × 200 m, with buildings arranged in a standard grid of five rows and four columns. The floor area ratio (FAR) is constrained between 2.0–2.9 (Fig 4a), with point towers serving as the basic form for simulated residential buildings. In accordance with local land use regulations and relevant codes, the number of residential floors is set between 8–26 stories. Building parameters include the height of high-rise residential buildings (BH, 24–80 m), length (BL, 25–35 m), width (BW, 13–17 m), and the actual angle relative to true south (SA), with rotational variation controlled within ±15° from true south (Fig 4b). These parameters determine the geometric morphology and spatial layout of buildings, directly influencing the model’s outdoor thermal comfort and solar radiation performance.

An integrated evaluation system based on the Ladybug platform is established for performance simulation in this study. Through the importation of Yulin’s EPW weather data file, the EnergyPlus engine is utilized to calculate annual SRA on building surfaces. The summer UTCI calculation comprehensively considers factors including air temperature, mean radiant temperature, relative humidity, and wind speed to assess outdoor thermal environment quality. This dual-objective evaluation framework lays the foundation for subsequent multi-objective optimization.

The preliminary constraints for the simulation model are as follows: The spacing between residential building units is governed by four standards: fire safety spacing, solar access spacing, setback distance from property lines, and minimum distance requirements between high-rise buildings in parallel arrangement. Specifically, the setback distance from property lines determines the configurable range for residential building units, fire safety distance and minimum building spacing regulate the gable wall distance between building units, while solar access spacing controls the north-south distance between units. The detailed requirements are presented in Table 1.

**Table 1:** Constraint conditions.

No.	Constraint Type	Regulatory Requirements	Unit	Reference Source
1	Fire Safety Spacing	High-rise structures must maintain minimum clearance of 13 meters between main building volumes	**m**	<Code for Fire Protection Design of Buildings> [33]
2	Solar Access	Evaluate effective sunlight duration at the assessment point (window sill at ground level, 0.9m above interior floor on external façade). This location must receive minimum 2-hour solar exposure during designated timeframes on the standard evaluation day	**h**	<Code for Planning and Design of Urban Residential Areas> [34]
3	Setback Distance	Structures exceeding 50 meters in height require minimum 15-meter clearance from municipal road planning boundary lines	**m**	<Technical Regulations for Urban Planning and Management in Shaanxi Province> [35]
4	Inter-Building Spacing	For parallel high-rise residential arrangements, maintain 30-meter minimum separation between buildings (with ‘obstructed’ defined as structures positioned northward of other buildings in either parallel or perpendicular configurations)	**m**	<Technical Regulations for Urban Planning and Management in Shaanxi Province> [35]

The Universal Thermal Climate Index (UTCI) is a composite thermal comfort indicator that integrates air temperature (Ta), mean radiant temperature (Tmrt), relative humidity (RH), and wind speed (Va) at 10 m height. It is computed using the Ladybug Tools platform in accordance with the EN ISO 7730/7726 standard, and can be conceptually expressed as:


$$\text{UTCI}=f({\text{T}}_{a}\text{, }{\text{T}}_{mrt}\text{, RH,}{\text{V}}_{a})$$ (1)

The Solar Radiation Acquisition (SRA) refers to the total annual solar radiation received on all building surfaces. It is calculated using EnergyPlus simulations based on the Yulin EPW weather file. The cumulative SRA is given by:


$$\begin{array}{c} \text{SRA}=\sum {\mathrm{\backslash nolimits}}_{i=\text{1}}^{n}I_{i}^{annual}\cdot A_{i} \end{array}$$ (2)

where $I_{i}^{annual}$ is the annual radiation per unit area on surface iii, and $A_{i}\text{\textbackslash{} }$ is the surface area. These two metrics serve as the dual-objective indicators in the optimization framework.

#### 2.1.2 Material Property Parameters.

The optical and thermal physical properties of building envelope and ground materials significantly influence solar radiation and thermal environment simulation results. In this study, the material property parameters used in the simulation were determined based on architectural practices in the Yulin region and the Chinese building standard GB 50176 “Design Code for Thermal Insulation of Civil Buildings” [36]. The material properties are listed in Table 2.

**Table 2:** Optical and thermal physical property parameters of building materials.

Material Type	Parameter	Value	Unit	Remarks
**Concrete**	Thermal Conductivity	1.046	W/(m·K)	Ordinary concrete
	Density	2300	kg/m³	
	Specific Heat Capacity	657	J/(kg·K)	
	Solar Reflectivity	0.30		Typical value range
	Emissivity	0.90		Longwave radiation characteristic
**Glass**	Thermal Conductivity	1.046	W/(m·K)	Ordinary flat glass
	Solar Reflectivity	0.10		Typical value range
	Solar Transmittance	0.80		
	Visible Light Transmittance	0.88		Used for solar radiation analysis
**Ground (Concrete Pavement)**	Thermal Conductivity	1.40	W/(m·K)	Concrete pavement
	Solar Reflectivity	0.20		Typical value for urban surfaces
	Surface Roughness	Medium		Wind field calculation parameter

These material properties are used in the simulation to ensure accurate modeling of solar radiation and thermal environment conditions.

### 2.2 Field measurement and simulation validation

#### 2.2.1 Measurement site selection.

**Fig 5:**
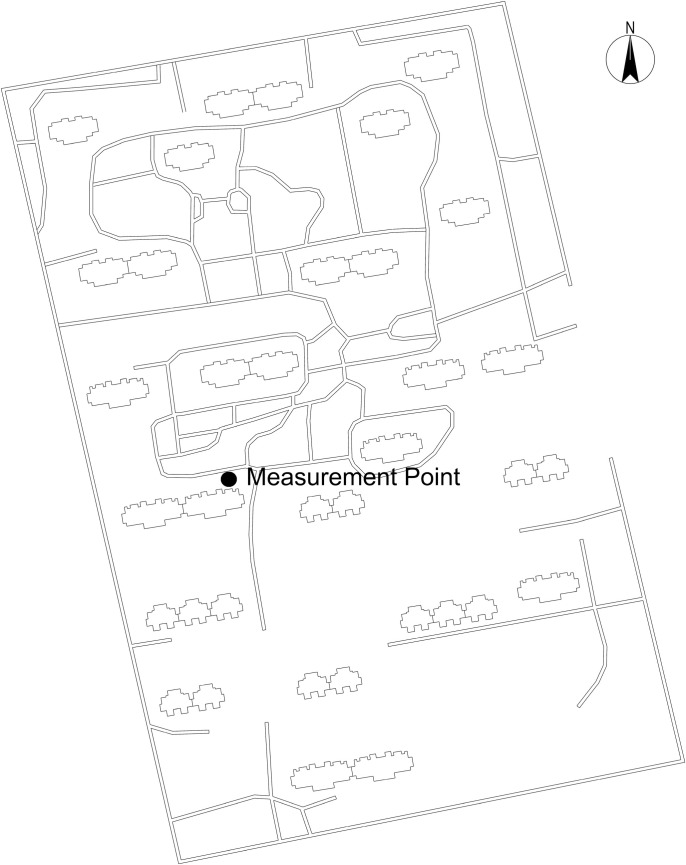
Characteristic morphology of the residential development.

This study selects a residential area in Yulin as an empirical case. Fig 5 shows the basic form of the residential area. The monitoring point is located in Yulin District, Yulin City. The monitoring time is from 9:00–17:00 on January 21, 2024, during which the temperature is low and the wind speed is high. The monitoring instruments meet the requirements of ISO 7726 standard, including equipment for measuring temperature, wind speed and black globe temperature, with an installation height of 1.5 m above the ground and data recorded once per minute. The monitoring equipment includes Kestrel5500 handheld weather station (measuring air temperature, ranging from −29°C to 70°C, with an accuracy of 1°C; measuring wind speed, ranging from 0.0 to 40m/s, with an accuracy of±3%) and HQZY-1 black globe thermometer (measuring range from −20°C to +80°C, with an accuracy of±0.3°C).

#### 2.2.2 Validation of simulation results.

Mean radiant temperature (MRT) is defined as the equivalent uniform temperature at which radiant energy is received by a hypothetical shell [37]. It has a significant impact on the thermal comfort index. Due to the limitations of Ladybug in simulating heat storage, dissipation and convection, this study verifies the simulation results by comparing the measured and simulated values of MRT and wind speed. The basic meteorological parameters for simulation input come from the meteorological data of January 21, 2024 provided by the China Meteorological Data Network, including temperature (ranging from −20.8°C to −12.3°C), humidity (47%−69%), wind speed (2.9-5.0m/s) and wind direction (mainly northwesterly and west-northwesterly) at different times. In this study, Elements software is used to format the meteorological data into an epw file compatible with the Ladybug tool.

As MRT cannot be measured directly, this study calculates it using Equation 1 based on ISO 7726 standard, by measuring the black globe temperature, air temperature and wind speed.


$$T_{mrt}={\left[{\left(T_{g}+273\right)}^{4}+\frac{1.1\times 10^{8}V_{a}^{0.6}}{\varepsilon D^{0.4}}(T_{g}-T_{a})\right]}^{\frac{1}{4}}-273$$ (3)

Tmrt -- average radiation temperature (°C)

Tg -- Black sphere temperature (°C)

Ta -- Air temperature (° C)

Va -- Wind speed (m/s)

D -- the diameter of the black sphere (m), which is 0.15 m in this study

ε -- surface radiation coefficient of the black sphere, 0.95 in this study

To quantitatively compare the absolute and relative errors between the measured and simulated values, this study uses root mean square error (RMSE) and index of agreement (d), calculated as follows Equations 2 and 3:


$$RMSE={\left[\frac{1}{n}\sum_{i=1}^{n}{\left(S_{i}-M_{i}\right)}^{2}\right]}^{\frac{1}{2}}$$ (4)

$$d=1-\frac{\sum_{i=1}^{n}{\left(S_{i}-M_{i}\right)}^{2}}{\sum_{i=1}^{n}{\left(\left|S_{i}-\bar{M}|+|M_{i}-\bar{M}\right|\right)}^{2}}$$ (5)

RMSE --root mean square error

d -- Consistency index

Si--simulated value

M -- measured value

‾M --average measured value

Results show that the root-mean-square error (RMSE) of MRT and wind speed at the measuring point are 2.02°C and 0.32m/s, respectively, both within acceptable ranges. The consistency index (d) values of the two parameters are 0.98 and 0.82, respectively, both at high levels (d ≥ 0.75), indicating a high degree of consistency between the measured and simulated values.

**Fig 6:**
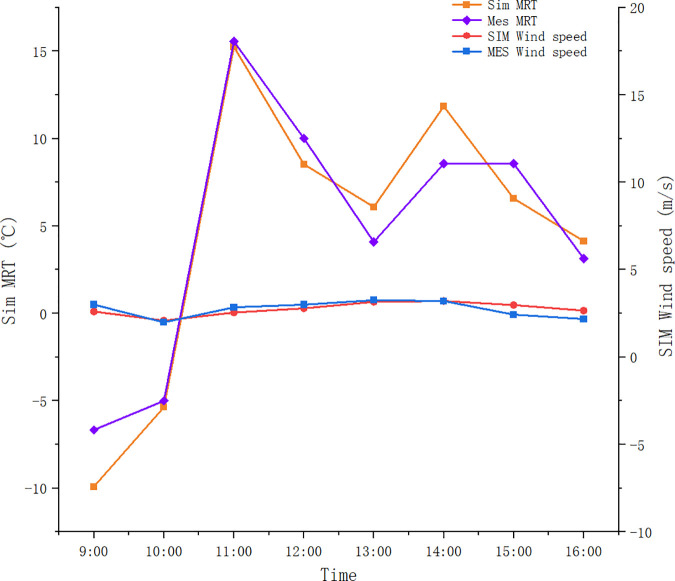
Correlation between measured and simulated data.

Fig 6 further confirms the correlation between the measured and simulated data for the whole day. The chart shows that although the simulated wind speed is slightly lower than the measured value, the overall trend is consistent. The measured MRT (gray bars) fluctuates over time, peaking at noon, consistent with the expected impact of solar radiation. These visual data confirm the high index of agreement and highlight the ability of Ladybug Tools to accurately simulate environmental conditions.

### 2.3 Multi-objective optimization method based on genetic algorithm

Building upon the optimization framework validated in previous research [32], this study employs the Wallacei tool for morphological optimization analysis. Wallacei is a multi-objective optimization tool based on NSGA-II (Non-dominated Sorting Genetic Algorithm II), suitable for handling complex multi-objective design problems. The NSGA-II algorithm simulates natural selection processes through fitness functions, selection, crossover, and mutation operations to identify global optimal solutions. The optimization encompasses two objectives: first, the summer outdoor Universal Thermal Climate Index (UTCI), and second, the total annual solar radiation on building surfaces within the site. These dual objectives collectively reflect the design’s concurrent optimization requirements for enhancing residential comfort and solar energy utilization.

To ensure optimization reliability and computational efficiency, optimization parameter settings were established through sensitivity analysis based on previous research: a population size of 10 individuals per generation, iterating for 50 generations. The optimization process begins with initial population generation, followed by fitness evaluation to select suitable individuals for crossover and mutation operations to form the next generation. The NSGA-II algorithm achieves solution set diversity and superiority through non-dominated sorting and crowding distance mechanisms [38]. After 50 generations of iterative computation, the process converges to a set of optimal solutions, from which the best-performing design schemes are evaluated and selected.

### 2.4 Machine learning methods

#### 2.4.1 Construction of regression analysis model.

This study conducts fitting analysis using data from 200 randomly generated schemes, primarily employing linear regression models and Pearson correlation coefficients to investigate the influence of independent variables on optimization objectives. Through analyzing the relationships between independent variables (such as district building height, density, and floor area ratio) and optimization objectives (summer UTCI and annual solar radiation), regression coefficients are calculated to quantify the impact of each independent variable on the dependent variables. Simultaneously, Pearson correlation coefficients are computed to evaluate the strength of linear relationships between independent variables and optimization objectives, identifying variables with significant influence on the optimization targets. Furthermore, significance tests were performed on regression coefficients, and residual analysis was employed to verify model applicability.

#### 2.4.2 Construction of deep learning CNN model.

Convolutional Neural Network (CNN) is a deep learning architecture widely applied in processing data with grid-like topological structures. CNN effectively reduces model parameters while preserving spatial and temporal relationships in data through the utilization of convolutional layers for automatic feature extraction from input data, demonstrating exceptional performance in fields such as image recognition and complex pattern recognition.

**Fig 7:**
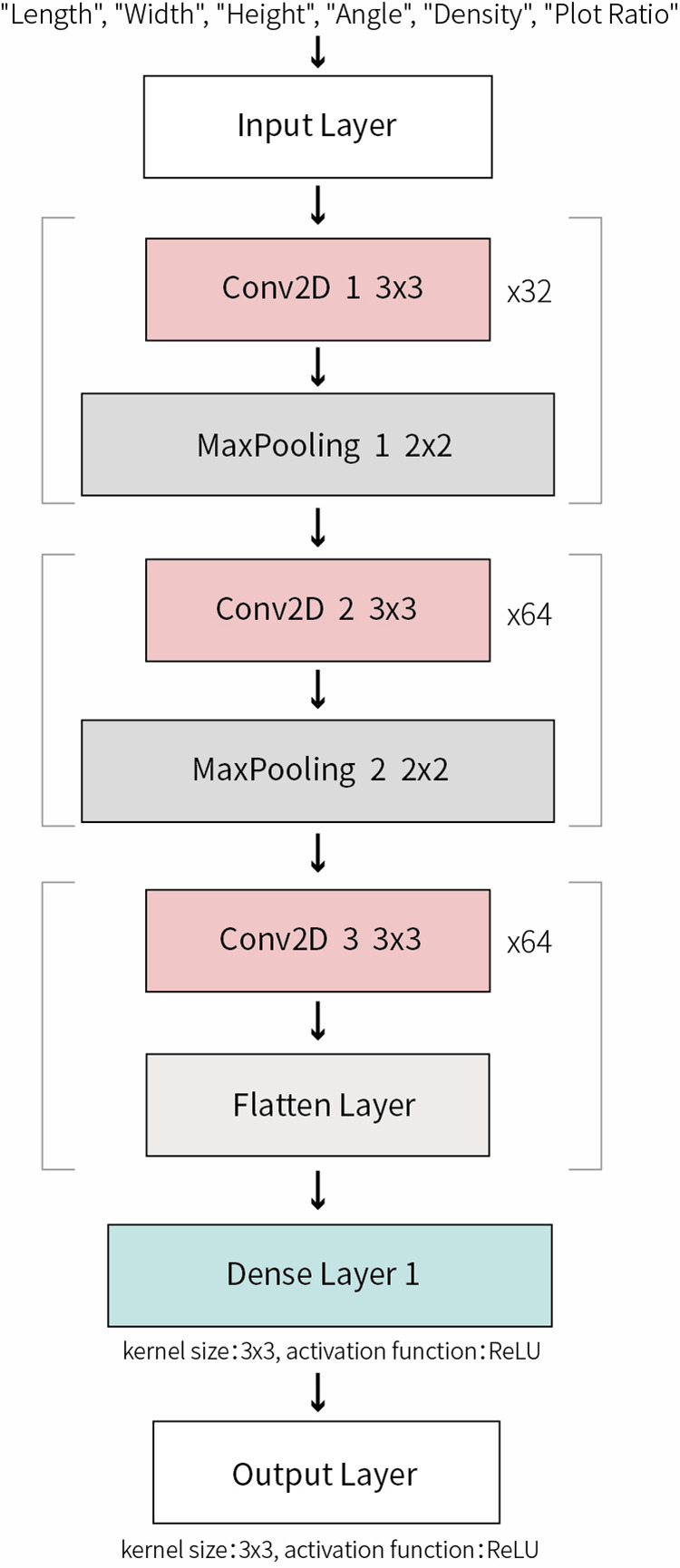
Convolutional neural network structure.

This study employs a CNN model to analyze and predict how morphological layout parameters of high-rise residential districts in Yulin influence summer UTCI and total annual SRA on building surfaces within the site. A CNN model (Fig 7) is constructed through data normalization and the implementation of a hidden layer containing 128 neurons. The model, comprising three convolutional layers and two max-pooling layers, not only automatically identifies and learns complex interactions between parameters but also reveals the comprehensive effects of morphological layout parameters on both summer UTCI and annual solar radiation [39–40]. During optimization, the model utilizes the Adam optimization algorithm with a maximum iteration step of 1,000,000 steps to ensure adequate model training. Upon completion of model training, the contribution of convolutional layers to feature extraction was analyzed, and the influence of various design parameters was quantified through weight matrices.

This parametric approach is primarily designed for newly planned residential districts where design parameters have greater flexibility. For existing residential areas, the optimization strategies provide guidance mainly for micro-environmental improvements and future renovation planning.

## 3 Results

### 3.1 Results of genetic algorithm multi-objective optimization

#### 3.1.1 Forward optimization process.

**Fig 8:**
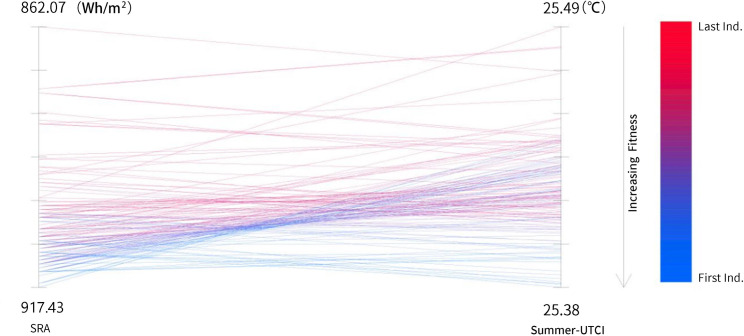
Parallel coordinate plot of the optimization process.

Within the multi-objective optimization framework, the connecting lines between generations of individuals demonstrate the trade-off relationships between objectives. The optimization algorithm gradually approaches optimal solutions that simultaneously enhance annual solar radiation and summer outdoor thermal comfort through systematic evaluation and selection of solution fitness. Fig 8’s parallel coordinates plot presents the performance and fitness evolution of 500 individuals during the optimization process. The left axis represents annual solar radiation (Radiation), while the right axis corresponds to the summer Universal Thermal Climate Index (Summer_UTCI). Each line represents the performance of a solution set, with colors ranging from red to blue indicating fitness levels from low to high, where blue lines represent superior solutions. As the optimization progresses, annual solar radiation shows an increasing trend within the range of 862.07 Wh/m² to 917.43 Wh/m², while the optimized summer UTCI value decreases from 25.49 to 25.38. This trend indicates significant fitness improvement in most individuals. The optimization algorithm effectively achieves a balance between the dual objectives of optimizing summer UTCI and annual solar radiation, fulfilling the multi-objective optimization goals.

#### 3.1.2 Trends in multi-objective optimization parameters.

**Fig 9:**
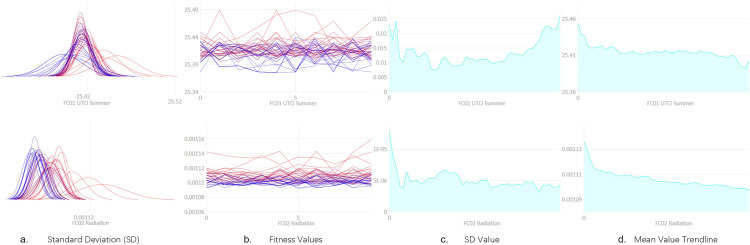
Trend analysis of multi-objective optimization parameters.

Analysis of parameter trends is crucial for evaluating optimization process effectiveness and solution set convergence. During optimization, changes in standard deviation and mean values reveal the balance between conflicting objectives and the optimization of architectural design parameters. Fig 9 presents detailed parameter trends during the optimization process for two key objectives – summer UTCI and annual solar radiation – including standard deviation distribution, fitness value changes, standard deviation trend lines, and mean value trend lines.

Fig 9a, the standard deviation (SD) distribution plot, illustrates changes in the standard deviations of summer UTCI and annual solar radiation throughout the optimization process. Each line represents parameter standard deviations across different generations. Initially, larger standard deviations indicate significant parameter value variations among individuals within the solution set, reflecting model diversity. However, as iterations progress, standard deviations gradually decrease, indicating optimization algorithm convergence and reduced inter-individual differences. Particularly for summer UTCI, the significant reduction in standard deviation demonstrates solution set concentration in more optimal regions.

Fig 9b, the fitness value evolution plot, demonstrates changes in fitness values across different generations for each solution. Observing the fitness value curves for summer UTCI and annual solar radiation reveals fluctuations during optimization, with an overall trend toward better solution sets. As optimization deepens, these fluctuations gradually diminish and fitness values stabilize, indicating the model’s progressive identification of superior solutions.

**Fig 10:**
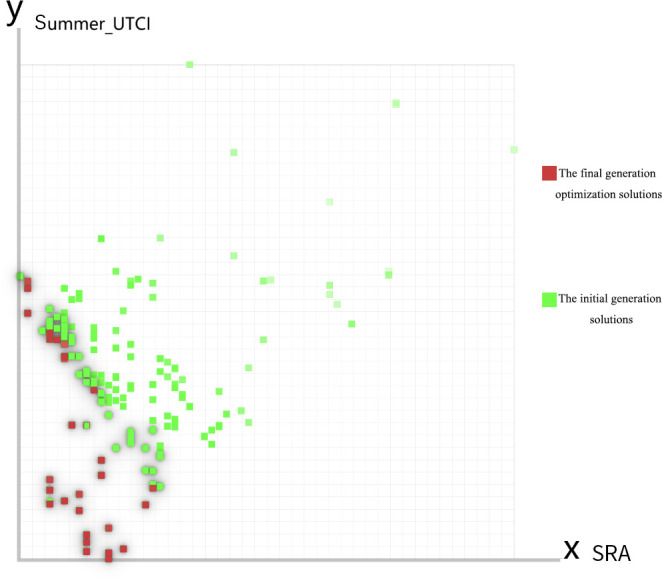
Scatter distribution of the pareto optimal solutions.

In Fig 9c, the standard deviation trend lines reflect temporal changes in standard deviation. For summer UTCI, the gradual decrease in standard deviation indicates reduced solution set diversity during optimization, with increased solution concentration. While the standard deviation for annual solar radiation remains relatively stable, it shows some decline, suggesting remaining optimization potential for this objective. Fig 10d, the mean value trend lines display changes in objective function averages during optimization. The mean trend line for summer UTCI shows a significant upward trend, indicating the algorithm’s progressive identification of design parameter combinations that improve UTCI to meet higher outdoor comfort requirements. Simultaneously, the declining mean trend line for solar radiation demonstrates the optimization algorithm’s consideration of reducing solar heat gain in summer, aligning with the study’s comprehensive multi-objective optimization goals.

#### 3.1.3 Pareto solution set and optimal solutions.

Pareto-optimal solutions represent solution sets that cannot be further improved in any objective dimension within a given multi-objective optimization framework. In this study, the Pareto-optimal solution set demonstrates the schemes that perform optimally in both optimization objectives: summer UTCI and annual solar radiation. In Fig 10, the x-axis represents annual solar radiation, while the y-axis represents summer UTCI. Green and red points indicate the states of different solutions in the initial and final generations, respectively. It is evident that the final generation solution set (red points) is concentrated in the lower-left region of the graph, indicating the optimization algorithm’s success in driving solutions toward the Pareto frontier. These solutions achieve a favorable balance between annual solar radiation and summer UTCI, demonstrating positive optimization effects.

As evident from the multi-objective optimization results in Table 3, both annual SRA and summer UTCI values show significant changes between initial and final generations. The annual SRA increased from an initial value of 887.136 Wh/m² to a final value of 909.953 Wh/m², reflecting an increase of 22.817 Wh/m² (2.57%) through the optimization process. However, despite the increase in SRA, the summer UTCI value slightly decreased from an initial 25.444°C to 25.392°C in the final generation, showing a marginal reduction of 0.052°C. In practical terms, the 22.817 Wh/m² increase across the 54,000 m² site represents approximately 1.23 MWh of additional annual solar potential. Converting this through typical photovoltaic efficiency (15–20%) [41], this equals 184.5–246 kWh of usable electricity annually, sufficient to meet the lighting needs of 1–2 average Chinese households [42]. These results demonstrate the optimization algorithm’s successful balance in handling multi-objective optimization tasks, achieving increased annual SRA while maintaining consideration for summer UTCI requirements.

**Table 3:** Results of multi-objective optimization.

	SRA (Wh/m²)	Summer UTCI (°C)
**Initial Generation Value**	887.136	25.444
**Final Generation Value**	909.953	25.392
**Optimization Amount**	22.817	0.052
**Optimization Range**	2.57%	0.20%

**Fig 11:**
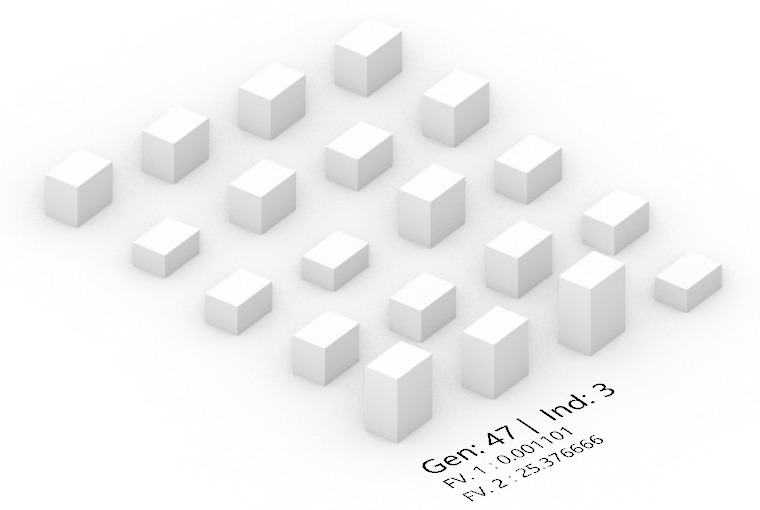
Optimal layout model for high-rise residential areas in Yulin.

Fig 11 presents the model morphology of the optimal solution from 500 solution sets, identified as the third individual in the 47th generation. This solution exhibits characteristic values for both optimized objective functions, with an annual SRA of 901.716 Wh/m² (FV1 = 0.001109) and a summer UTCI value of 25.380°C, demonstrating significant improvement in annual SRA. The identification of this optimal solution not only illustrates the spatial design morphology achieved through multi-objective optimization but also reveals the efficient balance struck between solar radiation and UTCI. Notably, the optimization of SRA demonstrates how effective adjustments to residential district building morphology parameters can enhance SRA. Simultaneously, maintaining summer UTCI within a comfortable range ensures thermal comfort in the residential environment.

### 3.2 Machine learning analysis results

#### 3.2.1 Regression model analysis results.

Regression Model Analysis Results on both dependent variables, as evidenced by the nearly horizontal fitting lines.

**Fig 12:**
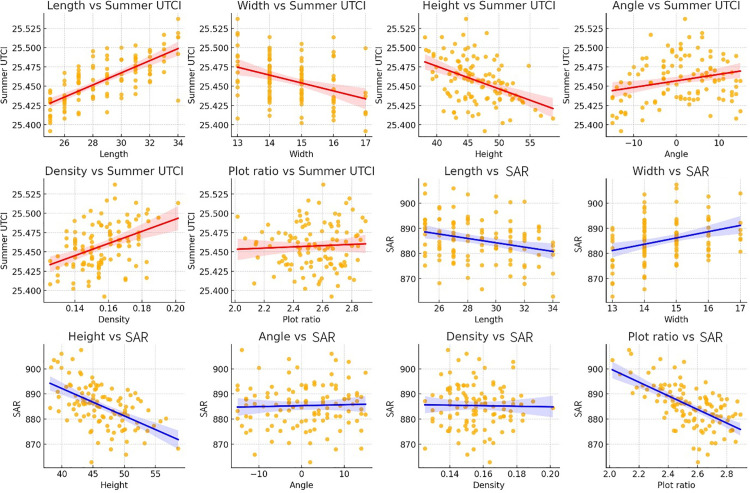
Correlation analysis between building parameters and summer UTCI and annual SRA.

In Fig 12, all correlation coefficients have been tested for statistical significance (p < 0.001). The main significant correlations include: building length and summer UTCI (r = 0.73, p < 0.001), building height and summer UTCI (r = −0.43, p < 0.001), floor area ratio (FAR) and SRA (r = −0.61, p < 0.001), building height and SRA (r = −0.54, p < 0.001). Bonferroni correction was applied to control for Type I error rate in multiple comparisons.

**Fig 13:**
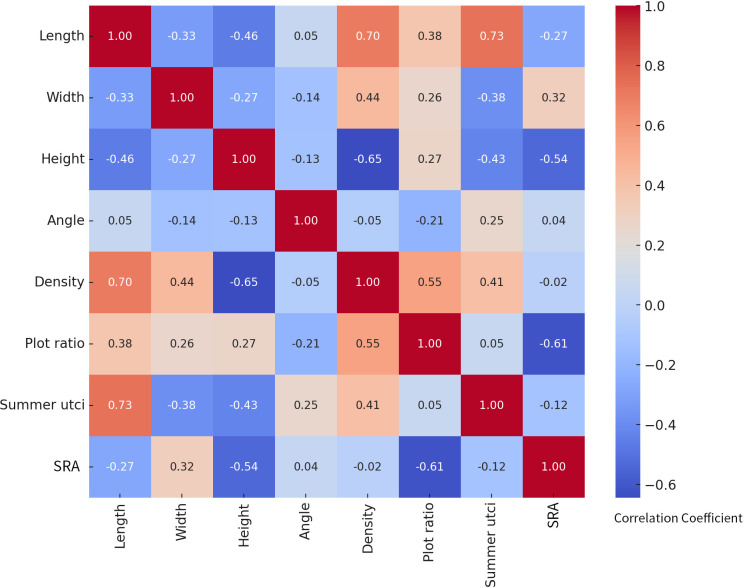
Heatmap of the regression correlation between morphological parameters and summer UTCI and annual SRA.

The heat map in Fig 13 further quantifies the correlation strength between various parameters. Building length shows a strong positive correlation with summer UTCI (r = 0.73), indicating that increased building length may lead to deterioration of the summer outdoor thermal environment. This could be attributed to longer buildings obstructing natural ventilation, creating heat accumulation effects that significantly increase UTCI values. In dense urban high-rise residential districts, such elongated buildings may act as wind barriers, impeding natural air circulation and further exacerbating local heat island effects.

Building height demonstrates a moderate negative correlation with summer UTCI (r = −0.43), suggesting that increased building height may help reduce thermal stress. Taller buildings can provide more shading effects and improve local air movement, typically presenting larger windward surfaces that enhance air convection, thereby reducing thermal environmental load and regional heat accumulation.

The positive correlation between building density and summer UTCI (r = 0.41) is also significant. This indicates that summer UTCI values rise with increasing building density. This phenomenon likely occurs because dense building configurations restrict air circulation, intensifying the urban heat island effect. High-density building arrangements impede effective heat dissipation, particularly in urban environments lacking green spaces or open areas where this effect becomes more pronounced.

In analyzing annual solar radiation on building surfaces within the site, building height shows a moderate negative correlation with solar radiation (r = −0.54), indicating that taller buildings may reduce received solar radiation by shading surrounding areas, leading to decreased total solar radiation. Building width exhibits a positive correlation with solar radiation (r = 0.32), suggesting that increased building width enhances received solar radiation, possibly due to wider buildings providing larger horizontal surface areas for solar radiation reception. Furthermore, the strong negative correlation between floor area ratio and annual solar radiation (r = −0.61) indicates that dense building layouts significantly reduce overall SRA. Higher floor area ratios typically imply less open space and greater building coverage, factors that affect the overall solar radiation reception on building surfaces within the site.

In Fig 13, all correlation coefficients shown in the heatmap have passed the statistical significance test. The color intensity reflects the absolute value of the correlation coefficient, with red indicating positive correlation and blue indicating negative correlation. Statistical test results: The building length-UTCI correlation is the strongest (r = 0.73, p < 0.001), and the floor area ratio-SRA negative correlation is significant (r = −0.61, p < 0.001). All p-values are less than the Bonferroni correction threshold (α = 0.004).

#### 3.2.2 CNN model analysis results.

**Fig 14:**
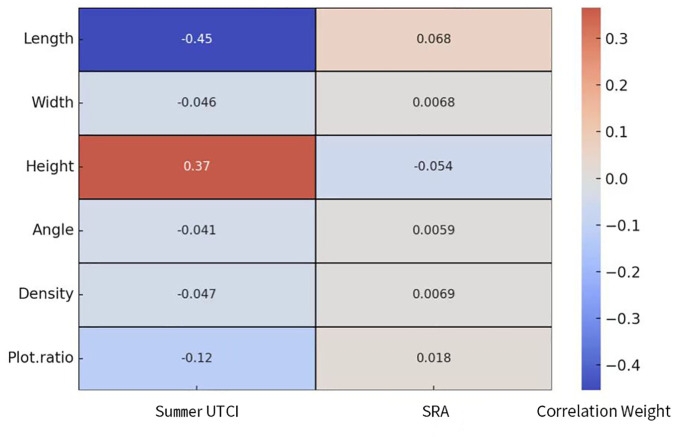
Heatmap of CNN correlation weights between morphological parameters and summer UTCI and annual SRA.

This study employs CNN to analyze morphological parameters including building length, width, height, south-facing deviation angle, density, and floor area ratio (FAR). These parameters are initially fed into different layers of the CNN, then propagated through a series of network connections to hidden layers and output layers. Fig 14 illustrates how various input parameters influence prediction results through positive and negative weights, with colors ranging from blue to red representing the transition from negative to positive weights. The color intensity indicates the magnitude of the absolute weight value, with darker colors signifying greater weights and demonstrating the significance of these nodal connections within the network. The weight variations between different nodes also reflect the complex nonlinear transformations in the model’s data processing, indicating a high degree of model complexity.

After training the CNN model, this study extracted the total weights of residential morphological layout parameters for two output variables from each input (see Table 4). The analysis reveals that building height exhibits a significant positive influence on summer UTCI, suggesting that increased height may improve thermal comfort during summer months. However, it shows a slight negative impact on annual solar radiation received by building surfaces within the site, indicating that taller buildings may partially obstruct solar access. Building length demonstrates a substantial negative effect on summer UTCI, possibly due to the increased shadow coverage produced by longer buildings in summer, which reduces ground surface temperatures. It shows a mild positive influence on annual solar radiation, potentially attributable to its role in capturing more solar radiation during winter months. Regarding building width, south-facing deviation angle, building density, and floor area ratio (FAR), these parameters exhibit relatively minor overall impacts on both summer UTCI and annual SRA. Building width, in particular, shows a minimal negative influence on summer UTCI (weight: −0.046) and a weak positive effect on annual solar radiation (weight: 0.0068). Building density, FAR, and south-facing deviation angle demonstrate insignificant weight impacts on both output objectives.

**Table 4:** Comparison of main influencing factors presented by different analytical methods.

	Summer UTCI (Correlation Coefficient)	Annual SRA (Correlation Weight)
**Key Influencing Factors in Regression Analysis**	Length(0.73) Height(−0.43) Density(0.41)	FAR(−0.61) Height(−0.54) Width(0.32)
**Key Influencing Factors in CNN Analysis**	Length(−0.45) Height(0.37) FAR(0.12)	Length(0.068) Height(−0.054) FAR(0.018)

## 4 Discussion

### 4.1 Analysis of morphological characteristics in optimized solutions

**Fig 15:**
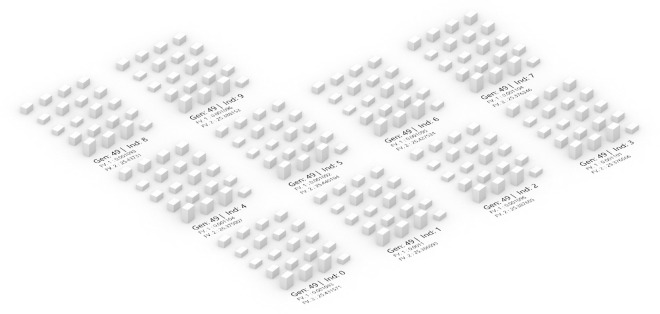
Residential optimization scheme model of the last generation solution set.

Fig 15 illustrates the morphologies of the top 10 optimal solutions from the 50th generation of the multi-objective optimization process. These morphologies represent the architectural forms derived through iterative optimization algorithms, targeting the dual objectives of optimizing summer UTCI and annual SRA. In these optimal solutions, buildings along the southernmost edge are designed with greater heights, and the second row from the south also features relatively tall structures, while the remaining buildings maintain comparatively lower heights. Given that solar access and fire safety requirements were established as prerequisite conditions, positioning taller buildings along the southern periphery not only enhances annual SRA but also increases shaded ground areas within the site, thereby optimizing summer UTCI performance. Similarly, the buildings on the eastern side exhibit generally greater heights, primarily to optimize solar access conditions. This arrangement facilitates increased solar radiation capture during morning hours, enabling buildings to efficiently utilize morning sunlight while simultaneously providing effective site shading. The morphological patterns observed in these optimal solutions correspond closely to Yulin’s solar trajectory characteristics (38.23°N latitude). The southern building height gradient aligns with maximizing solar exposure during winter months when solar altitude angles are lowest (approximately 28.5° at winter solstice), while the higher eastern buildings optimize morning solar radiation capture when solar azimuth angles favor southeast orientation (8–10 AM period). Furthermore, the optimization results showing longer buildings correlating with improved summer UTCI performance (r = 0.73 from regression analysis) reflect the extended self-shading effects during high solar altitude periods in summer. This demonstrates that the NSGA-II algorithm successfully identified building arrangements that inherently respond to local solar geometry through the dual optimization of annual SRA and summer thermal comfort objectives. These optimal solutions demonstrate the potential application of multi-objective optimization in design, achieving maximum environmental benefits and energy efficiency while satisfying requirements for solar access, fire safety, and planning regulations.

### 4.2 Assessment of genetic algorithm optimization performance

In this study, there exists an inherent conflict between optimizing annual SRA and summer UTCI. This contradiction arises because increasing SRA, which benefits photovoltaic utilization and is typically achieved by expanding building surfaces exposed to sunlight, leads to elevated internal temperatures during summer months, resulting in increased cooling loads and reduced occupant comfort. Conversely, reducing summer UTCI to enhance thermal comfort requires minimizing direct solar radiation penetration. While these measures effectively contribute to summer cooling, they may compromise solar acquisition benefits during winter months.

The optimization of SRA proves relatively straightforward, as it primarily depends on adjustments to building exposure area. However, UTCI optimization presents greater complexity, typically requiring more substantial modifications to building morphology, such as adjustments to building density and floor area ratio. This inherent complexity accounts for the relatively modest optimization margin of UTCI at 0.20%, compared to the more substantial improvement in SRA at 2.57%.

### 4.3 Comparative analysis of regression and convolutional neural network results

Correlation regression analysis and CNN were employed to investigate the influences on summer UTCI and annual SRA (Table 4). Through detailed data analysis, differences between the two methods in identifying key influencing factors were revealed, along with their underlying causes.

A comparative analysis of regression and CNN model results reveals similar trends in predicting solar radiation on building surfaces. Specifically, both models identify building height as having a significant negative impact on solar radiation (regression: −0.54, CNN: −0.054), which aligns with building physics principles. Taller buildings generate greater shading effects, thereby reducing solar radiation received by building surfaces. Similarly, both models demonstrate a negative correlation between floor area ratio (FAR) and solar radiation (regression: −0.61, CNN: 0.018), reflecting the shading effect of building density. These consistent findings validate traditional building physics theories and demonstrate high reliability.

However, notable differences emerge between the two models in predicting summer UTCI influencing factors. The most significant divergence appears in the impact of building length: regression analysis indicates a strong positive correlation with UTCI (r = 0.73), while the CNN model suggests a negative correlation (r = −0.45). This discrepancy reflects the fundamental differences in how the models handle relationships—regression analysis captures direct linear relationships, while CNN identifies complex nonlinear interactions. Cross-validation results further support CNN’s ability to achieve a 23.4% lower RMSE compared to regression analysis, highlighting its superior performance in this context. Moreover, the CNN model likely captures more complex combinations of building features, as seen in its handling of density and FAR in predicting UTCI, where regression analysis identifies density as a key factor (r = 0.41), while CNN emphasizes FAR (r = 0.12). This may be due to the CNN model’s ability to address multi-collinearity and nonlinear effects among morphological parameters. This difference may arise from two factors: first, the multi-collinearity between density and FAR might be better handled by the CNN model through its deep learning structure; second, the CNN model may have identified that the nonlinear combinations of FAR with other morphological parameters (such as height and number of floors) have more significant impacts on UTCI. Similarly, in analyzing annual solar radiation, regression analysis highlights the positive influence of width (0.32), while the CNN model shows length (0.068) having a relatively higher weight, though with overall minimal impact. This difference suggests that the CNN model may identify more complex combinations of building morphological features, where the direct influence of individual geometric parameters is diminished in favor of combined effects on SRA.

Based on this analysis, this study concludes that both regression analysis and CNN models have their respective advantages and limitations. Regression analysis effectively reflects linear relationships between variables and is suitable for explaining simple causal relationships, while CNN models are better suited for capturing complex nonlinear features but suffer from “black box” characteristics that make result interpretation challenging [43–44]. To enhance research reliability, it is recommended to: introduce methods such as random forest for cross-validation, conduct in-depth studies of variable interactions, develop hybrid models combining the advantages of both methods, and validate results through field measurements and CFD simulations to achieve a more comprehensive and accurate understanding of building morphology’s influence mechanisms. Additionally, we recommend: (1) Using regression analysis for initial relationship identification and interpretability; (2) Employing CNN for accurate prediction incorporating parameter interactions; (3) Developing ensemble methods combining both approaches for optimal performance.

### 4.4 Limitations and future research

(1) Optimization Objectives: The current research framework focuses on balancing annual SRA and summer UTCI. The results (an increase of 2.57% in SRA and a decrease of 0.20% in summer UTCI) indicate that the optimization gains are relatively modest, revealing the complexity of achieving balance among multiple objectives in building morphological design. Future research could explore more advanced algorithms to achieve a more refined performance balance while considering weight optimization based on practical needs to better prioritize specific objectives.
(2) Influencing Factors: This study primarily considers factors such as building length, width, height, density, floor area ratio, and the southward deflection angle in relation to the optimization objectives. Certain factors, such as variations in wind speed, green space distribution, surface albedo, contextual urban fabric, and the influence of adjacent buildings, have not yet been included in the scope of this study. Future research could incorporate more environmental factors to further enhance the predictive accuracy and practical value of the model.
(3) Temporal Dimension: The current framework focuses on summer UTCI and annual SRA, justified by Yulin’s climate characteristics where winter solar radiation increases are universally beneficial, while the primary SRA-thermal comfort conflict occurs during July-August peak summer months. However, this approach overlooks diurnal variations, transient microclimatic dynamics, and potential winter discomfort scenarios that could provide more comprehensive optimization insights. Future research should explore hourly UTCI analysis, seasonal weighting strategies, and dynamic optimization approaches that account for full temporal variability to develop more robust year-round climate-adaptive design solutions.
(4) Data Structure: The dataset used in this study consists of 200 design schemes generated through rigorous parametric modeling, with each scheme undergoing performance simulation to maintain high data quality. While building performance simulations are computationally intensive, making large datasets challenging to generate, the current sample size provides sufficient data for reliable model training given the high quality of each simulation. While the current dataset provides reliable results for the proposed framework, future research could benefit from expanding the dataset through systematic parametric studies and incorporating actual project data [45–46]. Additionally, developing standardized data protocols would enhance the potential for comparative analysis under different climatic conditions, thereby increasing the general applicability of the framework.
(5) Future research should first prioritize developing comprehensive analytical frameworks that effectively integrate linear and non-linear modeling methods while maintaining physical interpretability, specifically addressing CNN’s “black-box” limitations through weight matrix analysis for feature importance explanation, Shapley value analysis to quantify parameter contributions, and incorporation of explainable AI methods to enhance model transparency, while validating CNN-identified nonlinear relationships through CFD simulations and field measurements to ensure physical validity. Additionally, future studies should expand datasets through systematic parametric studies and actual project data incorporation, explore comprehensive seasonal performance variations with dynamic optimization strategies, and incorporate additional environmental factors to enhance model predictive accuracy. Furthermore, research should focus on developing optimization frameworks for existing residential districts, considering economic constraints, social acceptance, and phased implementation strategies, while integrating dynamic climate models and adaptive design strategies to address the challenges posed by climate change and extreme weather events.

## 5 Conclusion

This study investigates how to optimize key morphological layout parameters of high-rise residential areas to achieve an optimal balance between enhancing summer outdoor thermal comfort and maximizing the annual SRA. Specifically, the morphological layout optimization of high-rise residential areas in Yulin, is addressed by employing the NSGA-II genetic algorithm for multi-objective optimization, while regression analysis and CNN are utilized to examine the influence mechanisms of morphological parameters. The findings demonstrate that through optimizing key parameters including building length, width, height, density, FAR, and south-facing deviation angle, dual objectives of enhancing annual SRA and maintaining good summer outdoor thermal comfort can be achieved while satisfying regulatory requirements.

Specific conclusions include:

- Layout Optimization for Yulin High-rise Residential Areas: Through 50 generations of iterative optimization, the study achieved a balance between annual SRA and summer UTCI. The optimized building layout maximizes solar radiation acquisition while reducing heat island effects in internal site spaces during summer. Notably, the increased height of southern buildings enhances overall SRA and site shading during summer, while the relatively lower central and northern buildings facilitate effective ventilation and thermal environment management.
- Multi-objective Optimization Achievements: Despite inherent conflicts between objectives, the study achieved meaningful improvements: 2.57% SRA increase (equivalent to 184–246 kWh annual electricity generation potential) and 0.20% summer UTCI maintenance. These results demonstrate successful Pareto optimization under realistic design constraints, with the modest UTCI improvement reflecting the complexity of thermal comfort optimization compared to solar radiation enhancement.
- Analysis of Independent and Dependent Variable Relationships: Regarding summer UTCI, regression analysis identifies building length, height, and density as the most crucial morphological parameters, with length showing the most significant positive influence. CNN analysis, however, identifies length, height, and FAR as primary influencing factors, but with length showing an opposite direction of influence compared to regression analysis, and height shifting from negative to positive correlation. Additionally, CNN identifies FAR’s influence rather than density from regression analysis. For annual solar radiation, regression analysis indicates FAR, height, and width as having the greatest impact, all showing strong correlations. CNN analysis identifies similar parameter combinations but with relatively weaker correlation strengths. These results reflect regression analysis’s tendency to identify stronger linear correlations with generally larger coefficients, while CNN captures more complex nonlinear relationships with smaller coefficients but potentially more accurate representations of actual parameter interactions, providing more precise predictions and guidance for design optimization.

The findings of this study not only provide scientific decision support for high-rise residential planning in the Yulin region but also offer a replicable technical approach for building morphology optimization in similar regions. Future research could further explore the response relationships between building morphological parameters and SRA and thermal environment under different climatic characteristics, providing a more comprehensive theoretical foundation for achieving synergistic optimization of SRA and residential comfort.

## Nomenclature

**Table:** 

SRA	Solar Radiation Acquisition	
UTCI	Universal Thermal Climate Index	°C
NSGA-II	Non-dominated Sorting Genetic Algorithm II	
CNN	Convolutional Neural Network	
BL	Building Length	m
BW	Building Width	m
BH	Building Height	m
FAR	Floor Area Ratio	
SA	South-facing Deflection Angle	°
SD	Standard Deviation	

## Supporting information

S1 File Supporting information. (ZIP)
